# Subclinical responses in healthy cyclists briefly exposed to traffic-related air pollution: an intervention study

**DOI:** 10.1186/1476-069X-9-64

**Published:** 2010-10-25

**Authors:** Lotte Jacobs, Tim S Nawrot, Bas de Geus, Romain Meeusen, Bart Degraeuwe, Alfred Bernard, Muhammad Sughis, Benoit Nemery, Luc Int Panis

**Affiliations:** 1 Occupational and Environmental Medicine, Unit of Lung Toxicology, KULeuven, Herestraat 49 (O&N1 - box 706), 3000 Leuven, Belgium; 2 Centre for Environmental Sciences, Hasselt University, Agoralaan gebouw D, 3590 Diepenbeek, Belgium; 3 Faculty of Physical Education and Physiotherapy, Dept. Human Physiology & Sports Medicine, Vrije Universitiet Brussel, Pleinlaan 2, 1050 Brussels, Belgium; 4 Flemish Institute for Technological Research, Mol, Belgium; 5 Department of Public Health, Catholic University of Louvain, Belgium; 6 Transportation Research Institute, Hasselt University, Diepenbeek, Belgium

## Abstract

**Background:**

Numerous epidemiological studies have demonstrated adverse health effects of a sedentary life style, on the one hand, and of acute and chronic exposure to traffic-related air pollution, on the other. Because physical exercise augments the amount of inhaled pollutants, it is not clear whether cycling to work in a polluted urban environment should be encouraged or not. To address this conundrum we investigated if a bicycle journey along a busy commuting road would induce changes in biomarkers of pulmonary and systematic inflammation in a group of healthy subjects.

**Methods:**

38 volunteers (mean age: 43 ± 8.6 years, 26% women) cycled for about 20 minutes in real traffic near a major bypass road (road test; mean UFP exposure: 28,867 particles per cm^3^) in Antwerp and in a laboratory with filtered air (clean room; mean UFP exposure: 496 particles per cm^3^). The exercise intensity (heart rate) and duration of cycling were similar for each volunteer in both experiments. Exhaled nitric oxide (NO), plasma interleukin-6 (IL-6), platelet function, Clara cell protein in serum and blood cell counts were measured before and 30 minutes after exercise.

**Results:**

Percentage of blood neutrophils increased significantly more (p = 0.004) after exercise in the road test (3.9%; 95% CI: 1.5 to 6.2%; p = 0.003) than after exercise in the clean room (0.2%; 95% CI: -1.8 to 2.2%, p = 0.83). The pre/post-cycling changes in exhaled NO, plasma IL-6, platelet function, serum levels of Clara cell protein and number of total blood leukocytes did not differ significantly between the two scenarios.

**Conclusions:**

Traffic-related exposure to particles during exercise caused a small increase in the distribution of inflammatory blood cells in healthy subjects. The health significance of this isolated change is unclear.

## Background

Abundant evidence from epidemiological and experimental studies shows that traffic-related air pollution has adverse effects on the respiratory and cardiovascular systems[1-3]. A study in Europe attributed about half of all mortality from air pollution to traffic sources [4]. It is well-established that even daily average exposures to particulate matter can cause acute health effects [5,6]. Different studies of humans exposed experimentally in exposure chambers showed acute increases in both lung and systemic inflammation [7-11].

Studies investigating real-life exposure of participation in traffic also found significant associations with adverse health effects. Peters et al. [12] showed that "participation in traffic" was associated with the risk of developing myocardial infarction in the hour afterwards. McCreanor et al. [13] investigated exposure to traffic-related air pollution in a group of asthmatic subjects and found a greater reduction in lung function, when subjects walked for two hours along a busy street compared to walking in a park. On the other hand, Strak et al. [14] found only weak evidence for an association between exposure of cyclists during morning rush hour and changes in lung function and airway inflammation.

Commuters often find themselves in locations and situations where they are in close vicinity to busy traffic. They are thus acutely exposed to traffic exhaust, including ultrafine particles (UFP) from diesel engines. Exposure during commuting may therefore represent an important fraction of the daily exposure to air pollution. This is especially true for cyclists. Their higher minute ventilation leads them to inhale higher amounts of pollutants compared with sitting in a car [15,16]. On the other hand, commuter cycling is a convenient way to include moderate physical activity into the daily schedule [17]. Cycling to work is, therefore, promoted in the prevention of obesity and to offset the health impact of a sedentary life style. Moreover, cyclists do not pollute the air.

In other words, there is a contradiction between the messages pointing to the beneficial effects of exercising by cycling to work and those highlighting the adverse health effects of traffic-related air pollution [18]. To (partially) address this conundrum, we undertook a "real-life" exposure study in healthy volunteers. In a controlled experiment, non-asthmatic subjects cycled 20 minutes during two exposure scenarios: near a major bypass road with busy traffic (road test) and in a room with filtered air (clean room), and we assessed acute effects on lung and systemic inflammation.

## Methods

### Study population

All subjects taking part in the 'SHAPES injury surveillance system' [15] who had filled out at least two electronic diaries (n = 1048), received an e-mail asking if they were willing to participate in the field tests. Briefly, these participants had been previously asked to fill out questionnaires about bicycle-related traffic accidents and also to fill out a self-reported electronic diary for one year with details about travel frequency, time spent cycling and distance travelled. The inclusion criteria were: (1) age between 18-65 years; (2) having a paid job outside the home; (3) cycling to work at least twice a week; (4) living in Belgium. Two hundred eighty-one subjects were willing to participate. Of these 281 responders, after excluding smokers and those on anti-platelet therapy, 41 were chosen in chronological order and contacted personally by phone. Of the 41 recruited subjects, 38 took part in the experiments at both locations. The Ethics Review Board of the Medical Faculty of the Vrije Universiteit Brussel (VUB) approved the study. All subjects gave a written informed consent.

### Study design

Subjects performed two exercise trials during two exposure scenarios: cycling on a cycling track near a major bypass road in Antwerp (road test) and cycling in a room with filtered air (clean room), 12 to 29 days apart. The road tests were carried out over a five day period (from 4 May 2009 until 8 May 2009) with a mean of eight participants per day. The tests in the clean room were done on 11 days (between 18 May 2009 and 3 June 2009), with a mean of three participants per day. Test were carried out from 8 am until 17 pm. Persons were asked to come by train and had to walk 1200 meters from the train station to the location in Antwerp (road test) and 700meters to the location in Brussels (clean room test).

We measured the anthropometric characteristics of the participants and administered a questionnaire to collect information about their lifestyle and medication intake. A venous blood sample was drawn and exhaled NO was measured, before the exercise. After the exercise, participants rested for 30 minutes in a seated position followed by the post-cycling examination, which included exhaled NO measurement and a venous blood sample collection.

#### Cycling near a major bypass road (road test)

Participants cycled a pre-selected route in Antwerp on a dedicated cycling path parallel to a major bypass road (a very busy 10 lane motorway with up to 200,000 vehicles per day and a major flow of heavy duty diesel vehicles). The total trajectory is 5750 meters long and mostly situated between 10 and 100 meters from the edge of the motorway. The cycling path also passes over the motorway on a dedicated bridge. Participants were asked to cycle at about the same intensity and speed as their everyday cycling to and from work. They carried a heart rate monitor (Polar X-Trainer Plus, Polar Electro OY, Kempele, Finland) and the bicycle was equipped with devices to measure exposure to particles (GRIMM and P-TRAK) and a GPS.

#### Cycling in a room with filtered air (clean room)

To create a 'clean room', three devices were used simultaneously and continuously (i.e. 24 hours a day) during the whole testing period, in a laboratory located, in Brussels, at the VUB. The Bionaire^® ^Mini Tower air purifier (The Holmes Group, Inc., Milford, USA) was used to purify the air from particles down to one μm by HEPA filters. Fine particles were excluded from the ambient air with the MedicCleanAir^® ^(Häri-Prolectron AG, Bronschhofen, Switserland) and the Genano^® ^310 (Genano OY, Espoo, Finland). The MedicCleanAir device also removes indoor gases, including ozone, from ambient air. During the cycling test, on a cycloergometer in the laboratory, a steady state heart rate equal to the individual's mean heart rate measured during the road test, was obtained. The exercise began with an initial workload of 80 W for men and 50 W for women at a pedalling rate of 70-80 rates per minute. The load was gradually adapted to achieve the individual target heart rate during the first minute of exercise. The duration of the test was defined by the duration of the road test.

Fine and ultrafine particles, present in the direct surrounding of the participant, were constantly measured, using the same GRIMM and P-TRAK devices as used during the road test (see below).

### Exposure measurements

To measure personal exposure to particulate matter (PM_10_, PM_2.5 _and UFP) during the road test and during the clean room test, small portable, battery-powered, devices were used. The GRIMM 1.108 Dust monitor (Grimm Technologies Inc, USA) is a portable environment dust monitor that can simultaneously measure PM_1.0_, PM_2.5_, PM_10 _and total suspended particulate (TSP). Ultrafine particle counts at one-second resolution were measured using P-TRAK Ultrafine Particle Counters (TSI Model 8525, USA) for particles in the size range 0.02-1 μm (maximum 500,000 particles per cm^3^). The P-TRAK is a hand-held, field instrument based on the condensation particle counting technique using isopropyl alcohol. We refer to Berghmans et al. [19] and Int Panis et al. [15] for a detailed description of the PM and UFP measuring techniques while cycling in traffic.

To compare ambient PM_2.5 _PM_10_, NO, NO_2 _and ozone concentrations between the two exposure scenarios (road test and clean room), we compared these concentrations obtained from fixed measuring stations, close to the location of the road test and the clean room test.

### Clinical endpoints

#### Exhaled NO

Fractional exhaled NO was measured with an electrochemistry-based NIOX MINO device (Aerocrine, Sweden). This instruments complies with ATS/ERS recommendations [20]. The device contains a scrubber which makes the inhaled air NO-free. The procedure consists of maximal inhalation. Subjects were instructed to monitor a flow rate as visualized on a display to maintain a flow rate of 50 ml/second.

#### Blood collection and analysis

A non-fasting blood sample was collected in an EDTA tube (4 mL), in a tube containing 0.129 M (3.8%) sodium citrate (4.5 mL) and in a dry tube (5 mL). Blood cell counts and differential leukocyte counts were determined using an automated cell counter with flow differential (Cell Dyn 3500, Abbott, USA). IL-6 was measured on plasma samples with a commercially available ELISA (Human IL-6 Quantikine, R&D Systems, UK). Clara cell protein was measured on serum samples with an in house latex immunoassay, using the rabbit anti-protein 1 antibody from Dakopatts (Glostrup, Denmark) [21].

#### Platelet function

Platelet function was assessed (within one hour of sampling) with the Platelet Function Analyzer PFA-100 (Siemens Healthcare Diagnostics, USA) [22]. The PFA-100 test cartridge consists of a capillary, a blood sample reservoir and a membrane coated with collagen/epinephrine with a central aperture. Whole blood is aspirated through the capillary and the aperture, thus exposing platelets to high shear rates (5000/s) and to collagen and epinephrine, causing platelet activation. A platelet thrombus forms at the aperture, thus gradually diminishing and finally arresting blood flow. The time from the start of aspiration until the aperture completely occludes, i.e. the closure time, reflects platelet aggregation in a shear stress-dependent way.

### Statistical analysis

For database management and statistical analysis, we used SAS software (version 9.1; SAS Institute Inc., Cary, NC, USA). Non-normally distributed values were log-transformed. We used linear mixed-effects models with adjustment for temperature, relative humidity and heart rate to investigate pre-cycling vs. post-cycling measurement on the studied parameters. Because within-individual repeated measures of outcomes are correlated, random effects were estimated at the subject level. To study whether the change between pre/post-cycling measurements was different per exposure scenario, we included an interaction term between pre/post-cycling measurement and the exposure scenario (road test vs. clean room) in the model. We also ran the model with an interaction term between pre/post-cycling measurement and the UFP (or PM_2.5_) concentrations during cycling.

## Results

### Study population

Thirty-eight physically fit, non-asthmatic participants (26% women) with a mean age of 43 years (range: 28-58 years) and mean body-mass index (BMI) of 24 kg/m^2^, participated (Table 1). Five persons reported hay fever.

**Table 1:** Characteristics (n = 38)

	Mean (SD) or number (%)
Anthropometrics	

Men/women	28/10 (74%/26%)

Age, years	43 (8.6)

BMI, kg/m^2^	23.7 (3.1)

Lifestyle	

Former smoker	16 (42%)

Exposure to environmental tobacco smoke	3 (8%)

Regular alcohol use	20 (53%)

Medication use	

Antiplatelet medication	0 (0%)

Lipid-lowering medication	1 (3%)

Antihypertensive medication	3 (8%)

### Exposure measurements

The mean ambient air pollution (PM_10_), measured by the automatic monitoring network, was the same on the days of the road test compared with the PM_10 _concentration on days of the clean room test (24.4 vs. 23.3 μg/m^3^, p = 0.77). This was also the case for ambient PM_2.5 _concentrations (13.4 vs. 13.1 μg/m^3^, p = 0.68) and NO_2 _concentrations (30.2 vs 28.6, p = 0.15). However, NO concentrations were higher on days of the road test as compared with the NO concentrations on days that the clean room test was carried out (7.2 vs 5.4 μg/m^3^, p = 0.006) and ambient ozone level were lower (48.3 vs 63.7 μg/m^3^, p < 0.0001).

**Figure 1:**
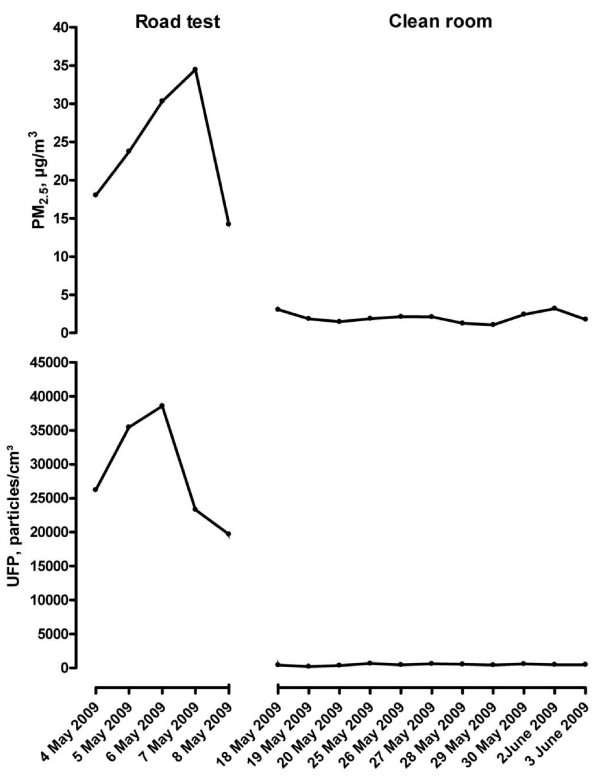
**UFP and PM_2.5 _concentrations**. UFP (bottom) and PM_2.5 _(top) concentrations on days of the study period during the road test (left) and in the clean room (right).

The average concentrations of particles to which the participants were exposed, during the road test and in the clean room are given in table 2. By design, concentrations of particles were higher during the road test (Figure 1). Average temperature was higher and relative humidity was lower in the clean room. By design the duration of cycling and the heart rate did not differ between the two exposure scenarios (road test and clean room) (Table 2).

**Table 2:** Exposure measurements during the road test and in the clean room

Endpoint	Road test	Clean room	p-value*
Average PM_10_, μg/m^3^	62.8 (23.6)	7.6 (3.3)	<0.001

Average PM_2.5_, μg/m^3^	24.2 (8.7)	2.0 (0.78)	<0.001

Average UFP, particles/cm^3^	28,867 (8479)	496 (138)	<0.001

Duration of cycling, min	20.8 (1.6)	20.2 (1.9)	0.20

Temperature,°C	15.2 (1.6)	21.6 (1.0)	<0.001

Relative humidity,%	57.0 (9.5)	45.7 (6.6)	<0.001

Heart rate, beats/min	131 (15.0)	131 (14.6)	0.90

% of maximal heart rate	74.0% (8.6)	74.1% (8.8)	0.90

Values are Mean (SD)

*Paired t-test

### Clinical endpoints

Baseline values (before cycling) of the clinical parameters were not significantly different between the road test and the clean room (Table 3). When we compared the clinical parameters pre/post-cycling (Table 4), separately for the two exposure scenarios (road test and clean room), we observed a decrease in exhaled NO of -4.4% change from baseline (p = 0.04) after the road test, but not in the clean room (-1.3% change from baseline; p = 0.63). However, the interaction term between pre/post-cycling measurement of exhaled NO and the exposure scenario was not significant (p = 0.38).

**Table 3:** Comparison of baseline values between the road test and the clean room

Endpoint	Road test	Clean room
Exhaled NO, ppb	29 (19 - 41)	24 (15 - 39)

PFA closure time, s	163 (135-197)	154 (125-176)

Plasma IL-6, pg/mL	1.47 (0.99-2.28)	1.53 (1.20-1.90)

Clara cell protein, μg/L	7.7 (5.6-11.5)	7.7 (5.6-10.3)

Blood leukocyte counts, per μL	4964 (1208)	4883 (1174)

Blood neutrophil counts, per μL	2937 (874)	2888 (884)

Percentage blood neutrophils,%	59 (8.0)	59 (7.1)

Data are geometric mean (25-75 percentile) for non-normally distributed variables or mean (SD) for normally distributed variables

Paired t-test: p > 0.08

**Table 4:** Percent change (pre/post-cycling) in endpoints per exposure scenario (road test or clean room)

Endpoint	Road test		Clean room		p-value for interaction		
	**Percent change (95%CI)**	**p-value**	**Percent change (95%CI)**	**p-value**	Exposure scenario*	**UFP^†^**	**PM_2.5_^‡^**

Exhaled NO,	**-4.4% (-8.3% to -0.37%)**	**0.04**	-1.3% (-6.5% to 4.1%)	0.63	0.38	0.63	0.50

PFA closure time	6.5% (-1.0% to 14.5%)	0.10	5.1% (-1.0% to 11.6%)	0.11	0.76	0.60	0.59

Plasma IL-6	17.4% (-6.7% to 47.9%)	0.18	-2.9% (-19.0% to 16.4%)	0.75	0.21	0.38	0.40

Clara cell protein	1.6% (-10.8% to 15.8%)	0.82	-0.27% (-11.7% to 12.7%)	0.97	0.90	0.91	0.80

Blood leukocyte counts	1.3% (-2.0% to 4.6%)	0.44	2.5% (-1.1% to 6.0%)	0.19	0.75	0.97	0.71

Blood neutrophil counts	**4.6% (0.48% to 8.7%)**	**0.04**	2.4% (-2.3% to 7.2%)	0.32	0.36	0.35	0.20

Percentage blood neutrophils	**3.9% (1.5% to 6.2%)**	**0.003**	0.22% (-1.8% to 2.2%)	0.83	**0.004**	**0.02**	**0.01**

Analysis adjusted for temperature, relative humidity and heart rate

*p-value for the interaction between pre/post-cycling measurements and exposure scenario (road test or clean room)

^†^p-value for the interaction between pre/post-cycling measurements and UFP concentrations

^‡^p-value for the interaction between pre/post-cycling measurements and PM_2.5 _concentrations

Platelet function, IL-6, Clara cell protein and total leukocyte counts did not change significantly from baseline after cycling, neither during the road test, nor in the clean room. However, the percentage of blood neutrophils (though not their absolute number) increased by 3.9% after cycling in the road test (p = 0.003), but not in the clean room (0.22%; p = 0.83). A significant interaction (p = 0.004) between percentage of neutrophils and exposure scenario was observed.

In a model that included either UFP counts or PM_2.5 _concentrations, the interaction terms were only significant for the percentage blood neutrophils and not for the other measured parameters (Table 4).

Similar results were obtained if we excluded the five persons that reported hay fever from our analysis.

## Discussion

In subjects free of lung and cardiovascular disease, a small, immediate (30 minutes after moderate exercise) increase in the percentage of blood neutrophils was observed in response to cycling in traffic-related exposure. Platelet function and a biomarker of lung permeability (Clara cell protein) did not show rapid changes between pre/post-cycling measurements in either exposure scenario. The change in pre/post-cycling measurement of exhaled NO did not differ significantly between the two scenarios.

NO is expressed by different cell types in the respiratory tract including epithelial cells, macrophages, neutrophils, mast cells and vascular endothelial cells [23]. Previous studies found that exhaled NO can be positively associated with exposure to air pollution both in healthy adults [24-26] and asthmatic subjects [27]. Proinflammatory cytokines can induce iNOS (inducible nitric oxide synthase), which releases large quantities of NO several hours after exposure [23]. In our study, exhaled NO was measured already 30 minutes after exposure and we observed a decrease after cycling in polluted air . It is known that smokers have lower levels of exhaled NO, compared with non-smokers [28-30]. Exposure to environmental tobacco smoke is also associated with decreases in exhaled NO [28,31-33]. The mechanisms for this decrease are unclear. Accelerated uptake of NO, inactivation of NO by oxidants, increased breakdown of NO or damage to NO producing epithelial cells by toxins may play a role in the observed decrease of exhaled NO caused by active or passive smoking [32].

Besides effects in the lung, we also looked at systemic inflammation by measuring total and differential blood leukocyte counts and plasma IL-6. We observed a significant increase in the percentage of blood neutrophils after cycling in polluted air, but not in filtered air. No changes occurred in plasma levels of IL-6. Neutrophils are known to be involved in inflammatory processes. It has been shown that neutrophils are increased after exposure to diesel exhaust [34].

We did not observe a significant increase in serum Clara cell protein levels after cycling in polluted air. An experimental animal study, with exposure to diesel exhaust, enriched with concentrated PM_2.5 _did not result in elevated serum Clara cell levels in the blood of rats [35]. Increased serum Clara cell levels have been associated with recent changes in PM_2.5 _in elderly [36] and have also been reported in cyclists after 2 h of cycling during an ozone episode [37].

There was a substantial difference in exposure to ultrafine particles between cycling during the road test and cycling in the clean room. However when we compare our values of ultrafine particles counts (28,867 per cm^3^) with those of other studies, we must conclude that exposure in our study was not very high. In Oxford Street, concentrations of PM_2.5 _were similar (median: 28 μg/m^3^; range 14 to 76 μg/m^3^) while concentrations of PM_10 _(median 125 μg/m^3^; range 62 to 161 μg/m^3^) and ultrafine particle counts (median 64,000 per cm^3^; range 40,000 to 92,000 per cm^3^) were about twice as high than in our experiment. On the other hand, in that study [13], even in Hyde Park, exposure was higher than in our study which used filtered air as a control situation. Strak et al. [14] found a median exposure in the high polluted traffic route of 41,097 particles/cm^3^. In addition, the mean duration of cycling was 20 minutes in our experiment, which is a realistic time, comparable with commuting from home to work. Similar experiments [13,14] have used exposure times of up to two hours, which is six times longer than in our study. Combined with the lower exposure concentrations, this means that the total exposure to UFP in our experiment was about an order of magnitude lower than in the few similar studies of the literature [13,14]. Moreover, our study included only healthy, trained cyclists. It is, therefore, remarkable that even at these low exposures, we were able to detect significant changes, although it might explain the fact that we only see minor changes in the measured health parameters.

The exercise during the road test and the clean room test was tightly controlled, thus minimizing bias. In the clean room the heart rate and the duration of cycling was the same as during the field test. These conditions, together with the fact that subjects had no other exposure after cycling and before the second blood sampling, strengthen the causality of the preclinical changes in association with traffic-related exposure in healthy volunteers. On the other hand, it is not clear whether these observations imply long-term negative health outcomes in regular commuter cyclists. Strong evidence exists that regular physical activity contributes to the prevention of chronic conditions and that it is associated with a reduced risk of premature death [38-40].

This study has limitations. The number of participants was rather small. Test persons were healthy, experienced cyclists, because we wanted to test real-life exposure in commuters. But therefore, observed effect could be underestimated. Health effects were measured very shortly after exposure (after 30 minutes). Therefore effects that appear later, could have been missed. On the other hand, the short time-frame between exposure and post-measurement minimizes other exposure after cycling. Pollen or aeroallergen levels were not taken into consideration. Finally, the conditions during the road test and in the clean room are not entirely comparable. Although we tried to minimize bias between the road test and the clean room test, by tightly controlling exercise (e.g heart rate and duration of cycling), we did not take into account noise and stress levels persons were exposed to during the road test.

It is not unusual for health promotion messages to face contradictions or ambiguities when promoting one measure to benefit health (e.g. daily cycling) while potentially increasing the risk of an adverse effect (e.g. increasing exposure to air pollution). This may pose problems of scientific credibility and/or acceptance of the health education messages. For instance, smokers are sometimes reluctant to stop smoking because they will gain weight, which is also a risk factor for premature death. Similarly, promoting breast feeding to protect infants against infections and perhaps future allergy, also leads to a high exposure of the infant to lipid-soluble pollutants (e.g. persistent organic pollutants, such as DDT, PCBs, dioxins) [41,42]. Promoting the regular consumption of fish to increase the intake of polyunsaturated fats may also lead to exceed the intake of PCBs [43]. In general, the consensus is that the beneficial effects of smoking cessation, breast feeding, or a balanced diet outweigh the hypothetical negative side effects, at the individual level. However, this does not mean that public health efforts should not be directed at minimizing the adverse effects.

## Conclusion

In conclusion, we observed in healthy cyclists, a small, immediate (30 minutes after moderate exercise) increase in the percentage of blood neutrophils. We do not believe, based on our limited findings, that healthy people should be discouraged from cycling to work in heavy traffic (provided it is safe), although from a public health point, cycling tracks should be developed away from busy roads. More importantly, traffic-related pollution should be decreased.

## List of abbreviations

UFP: ultrafine particles; IL-6: interleukin-6; NO: nitric oxide; VUB: Vrije Universiteit Brussel; GPS: global positioning system; PM_10_: particulate matter with a diameter of less than 10 μm; PM_2.5_: particulate matter with a diameter of less than 2.5 μm; EDTA: ethylenediaminetetraacetic acid; PFA: platelet function analyzer; BMI: body-mass index; O_3_: ozone; iNOS: inducible nitric oxide synthase; DDT: dichlorodiphenyltrichloroethane; PCBs: polychlorinated biphenyls

## Competing interests

The authors declare that they have no competing interests.

## Authors' contributions

TSN, LIP, BdG and RM participated in the design of the study. LJ drafted the manuscript, performed the statistical analysis with help of TSN and BN, and collected the data with help of MS and performed the biochemical analysis (with exception of Clara cell). AB analysed the Clara cell protein in serum. BD was responsible for the particulate measurements. All authors read and approved the manuscript.
